# “I always tried to forget about the condition and pretend I was healed”: coping with cervical cancer in rural Ghana

**DOI:** 10.1186/s12904-018-0277-5

**Published:** 2018-02-12

**Authors:** Charity Binka, Samuel Harrenson Nyarko, Kofi Awusabo-Asare, David Teye Doku

**Affiliations:** 1 School of Public Service and Governance, Ghana Institute of Management and Public Administration, Achimota, Accra, Ghana; 2 Department of Population and Behavioural Sciences, School of Public Health, University of Health and Allied Sciences, Hohoe, Ghana; 3 Department of Demography, College of Public Policy, University of Texas at San Antonio, San Antonio, Texas USA; 4 Department of Population and Health, University of Cape Coast, Private Mail Bag, University Post Office, Cape Coast, Ghana; 5 Faculty of Social Sciences, Sciences, Health Sciences, FI – 33014 University of Tampere, Tampere, Finland

**Keywords:** Coping, Strategies, Cervical cancer, Patients, Rural Ghana

## Abstract

**Background:**

Cervical cancer is a very common disease among women in Ghana and in the world as a whole. However, there is a dearth of information on the mechanisms cervical cancer patients adopt to cope with the condition in Ghana. This study sought to explore the strategies adopted by cervical cancer patients in rural Ghana to cope with the disease.

**Methods:**

In-depth interviews were conducted to collect qualitative data from cervical cancer patients in a health facility in the Volta Region of Ghana. Data processing was done using the R software package for Qualitative Data Analysis (RQDA) and a thematic approach was used to analyse and present the results.

**Results:**

The results show that cervical cancer patients adopted personal and psychological strategies such as sexual abstinence, personal hygiene, and disease denial to cope with the condition. Respondents also described social, financial and non-material support services they received from family members and the church as critical resources, which helped them to manage the conditions of the disease. Respondents also reported that faith healing, herbal and orthodox medicines helped them to manage the symptoms of the disease.

**Conclusions:**

Cervical cancer patients used a variety of coping strategies to manage the disease. Yet, it will be essential for interventions to focus on strengthening knowledge about the disease. This study underscores the need for financial, social and material support as well as an encouragement of the use of health services among cervical cancer patients.

## Background

Cervical cancer is the fourth most common cancer in women and accounts for a significant proportion of disability and deaths in the world [1]. It is estimated that there were about 528,000 new cases of cervical cancer worldwide and an estimated 266,000 mortalities in 2012 alone with the majority of the global burden occurring in the developing world [1]. Lack of access to screening and treatment services contributes significantly to cervical cancer-related deaths that occur among women living in low and middle-income countries [2]. Chronic illnesses including cervical cancer have a significant impact on the physical, functional, emotional, social and spiritual well-being of patients [3].

Generally, the early stage of cervical cancer may not show any noticeable signs or symptoms. The possible signs and symptoms patients may notice include vaginal bleeding after sex, in-between menstrual periods or after menopause [4, 5]. However, in most patients, there may be no symptoms at all whatsoever until the disease is at the advanced stage [4, 5]. At this stage, other symptoms may include vaginal pain especially during sex, unpleasant-smelling vaginal discharge, vaginal discharge stained with blood, abnormal heavy bleeding from the vagina, and change in menstrual cycle that cannot be explained as well as pain when passing urine [4]. In the advanced stage where the disease has spread into neighbouring tissues, it can trigger additional symptoms such as constipation, blood in urine, urinary incontinence, bone pain, swelling of legs and tiredness or loss of appetite [4]. The fact that these signs and symptoms do not manifest until the disease has reached advanced stage has severe implications for the wellbeing of women.

Studies have established that people adopt various coping strategies to manage stressful situations associated with chronic diseases such as cervical cancer, which may affect their psychological, physical and social well-being [6, 7]. Coping strategies are the specific efforts, thoughts, and behaviours that are used to manage the internal and external demands of situations that are appraised as stressful [8, 9]. It has been observed that the epidemic of chronic illness is steadily moving towards crisis proportions, yet maintaining or enhancing the quality of life of individuals living with such situations has not been given the attention it deserves [10]. In addition, information on coping strategies for a chronic illness such as cervical cancer is limited globally and in Ghana as well. Consequently, this study sought to explore the coping strategies adopted by cervical cancer patients in a rural community in Ghana in managing the condition.

## Methods

### Study setting and design

This study was conducted at the Battor Catholic Hospital in the North Tongu District of the Volta Region in Ghana. It is the only health facility that provides cervical cancer screening and treatment in a rural environment in the country. A qualitative research method was adopted for this study. The qualitative approach was used because it helped to explore and understand the motives, meanings, reasons and other subjective experiences that informed the actions of the respondents [11].

### Study population

The population of the study comprised all women who reported at the gynaecology department of the health facility and had been diagnosed with cervical cancer and had survived the disease. The respondents were recruited based on the following criteria: whether they were diagnosed with cervical cancer by the Gynaecology unit of the health facility; women who were 30 years and above, and willing to participate in the study. The age range was in line with the cervical cancer guideline, which recommends that every woman from age 30 to 49 should at a minimum have cervical cancer screening at least once in her lifetime [2]. Furthermore, it has been established that the prevalence of Human Papilloma Virus (HPV), which is recognised as the major risk factor for the disease, is highest among women in their reproductive age and beyond, followed by post-menopausal women [12]. The respondents included those who were in the early stage of the disease and those who had undergone surgery or treatment. Patients who were in a life-threatening condition and those who had undergone a total hysterectomy at the time of the study were excluded from the study, as they were too unwell to be able to participate in the research.

### Sampling procedure

The sampling technique adopted was purposive. Only women who have survived cervical cancer or in the early stage of the disease were considered for the purpose of the study. In recruiting respondents, the records of patients were reviewed at the registry of the Gynaecology Department of the health facility to identify patients who had been diagnosed with the disease. In total, 60 patients were identified from the hospital register at the time of the study. The patients were from various parts of Ghana. Afterwards, phone calls were made to patients to seek their participation in the study. Many could not be reached because they were either dead or had wrong telephone numbers recorded in the register. Some of the patients contacted declined to participate in the study because they considered their disease condition as a private matter. Consequently, only 15 women agreed to participate in the study and were recruited. Nevertheless, a thematic saturation was achieved after interviewing the 15 respondents.

### Data collection

An in-depth interview guide was developed to collect data from the respondents. The interview guide comprised issues on the background characteristics of respondents, personal coping strategies, forms of support and health-seeking behaviour of the respondents. Respondents were interviewed in the following Ghanaian languages respectively: Ewe, Twi, and Dangme. Three female research assistants, who were fluent in the three languages, were recruited and trained on the contents of the data collection instrument and how to effectively conduct the interviews to ensure data quality and without compromising ethical issues.

Due to the sensitive nature of the subject, interviews were conducted in secluded places, which respondents considered most comfortable to them in order to ensure respondents’ privacy and confidentiality. To ensure the accuracy of data collected, the researchers and field assistants immediately conducted checks with the respondents after the interviews to verify their trustworthiness. The interviews were also peer-reviewed by the research team.

### Data processing and analysis

The interviews were tape-recorded and transcribed in English by research assistants who were eloquent native speakers of the local languages and competent in the use of the English language. The transcripts were then given to language experts in the three local languages to review and revise the content to ensure the accuracy of findings. The transcribed interviews were coded and processed with the R software package for Qualitative Data Analysis (RQDA) (version R-3.2.2). A thematic approach to qualitative data analysis was, therefore, applied [13]. This involved generating deductive codes that emerged from the interviews. The results were presented in quotations from the respondents to support the contextual analysis and provide an opportunity for discussion of the results of the study.

## Results

### Background characteristics of respondents

A summary of the background characteristics of the respondents is presented in Table 1. Most of the respondents were 50 years and above,  had secondary school education, were married women and were self-employed. The majority (12) of the respondents had four and an above number of children and they were all Christians. The majority also neither smoked tobacco (14) nor took alcoholic drinks (11). The youngest age at first sexual intercourse was reported as 15 years, while the oldest was 28 years, with the average age at first sexual intercourse being 17.

**Table 1:** Background characteristics of respondents

Variable	Number of respondents
Age group	
30–39	1
40–49	4
50–59	6
60+	4
Level of formal education	
None	1
Primary	4
Secondary	8
Tertiary	2
Marital status	
Married	10
Divorced	2
Separated	2
Widowed	1
Employment status	
Unemployed	3
Self-employed	10
Housewife	2
Number of children	
1–3	3
4–6	9
7 +	3
Religious affiliation	
Christian	15
Smoking status	
Yes	1
No	14
Drinks alcohol	
Yes	4
No	11

### Coping strategies among respondents

The broad themes that emerged from the interviews comprised personal coping strategies, forms of support and health-seeking behaviour.

### Personal coping strategies

The respondents reported adopting a number of active personal coping strategies including disease denial and positive attitude, abstinence from sex and personal hygiene.

#### Disease denial and positive attitude

Respondents employed active psychological coping strategies such as denial of the disease and positive thinking to manage the stressful conditions associated with the disease. Some respondents indicated that they coped with the pains and the fears they experienced. They also behaved in a way to make people believe that the condition no longer existed:

*“To cope, I always tried to forget about the condition (cervical cancer) and pretend I was healed.”* (Respondent 6).

One respondent also had to comport herself and tried as much as possible to remain level-headed in the midst of the conditions of the disease. According to her, handling the situation calmly and putting up a positive attitude helped prevent the condition from worsening and facilitated her healing process. The statement below presents the experience of one of the respondents:

*“I tried to handle the situation calmly in order to be able to cope with the condition*” (Respondent 9).

#### Sexual abstinence

Respondents further reported that they adopted these behaviours to manage the pains and blood flow that are associated with the disease. For the sexual abstinence, some of the respondents reported that they withdrew from sexual activity due to their prior experience with bleeding after sexual intercourse The respondents explained that they were actively engaged in sexual activity before the diagnosis but had to decide with their partners to abstain from sexual intercourse in order to stop the blood flow and reduce the pains which they were experiencing. The following statement was reported by a respondent:

*“During treatment (cervical cancer treatment), it was not easy having sexual intercourse. The disease came with painful sores; so until the treatment was completed and the sores healed, I had to abstain from sex”* (Respondent 2).

Another respondent also made a similar report concerning her sexual abstinence with her partner:

*“After going through the treatment process (cervical cancer treatment), I was afraid that if I engage in any sexual activity, I might start bleeding. So I have even taken sex off my mind”.* (Respondent 14).

#### Personal hygiene

Respondents further reported adopting personal hygiene as a way of managing the condition. One personal hygiene strategy adopted by the respondents was the consistent use of sanitary pads, which they bought with financial support from family and friends. Those who could not afford the sanitary pads resorted to the use of old cloth to control the leaking blood. For instance, one respondent reported:

*“Just like how we usually use the sanitary pads during our menstrual period, that was how I managed myself. This is to make sure that whenever I went out to a place or sat in a public place, I would not soil myself”.* (Respondent 1).

Respondents also reported using disinfectants to prevent odour from the blood flow. Some respondents indicated that they added disinfectants such as Dettol to water to clean themselves and their vagina regularly. They explained that using disinfectants to clean their vagina would help kill the germs and bacteria they might have attracted from the environment due to the blood flow. A respondent said:

*“After bathing, I always dropped some Dettol (disinfectant) in water and clean my vagina with it, then I put on some sanitary pad. I keep changing the sanitary pad from time to time so that I do not soil myself.”* (Respondent 11).

### Forms of support

Respondents were asked to indicate the types of support they received, which helped them to cope with the condition. The respondents identified three broad types of support: social, financial and spiritual support. They indicated that these supports were from their spouses, religious leaders, church members, children, friends and various associations, which they belong to.

#### Social and spiritual support

Some respondents reported that they were supported socially and spiritually by their families, friends and the church. This, they did as believers in God, who are obliged to help the needy and the poor. One respondent said:

*“My children, my sister, and my sister’s children all cared for me very well. They did not want me to be depressed about the condition because they thought I may die out of depression”* (Respondent 5).

One respondent also reported the social support she received during the treatment process. Her sister had been running all the required errands during the treatment process for her. She reported that these include queuing for treatment, picking the folder at the hospital, escorting her to the laboratory, picking her medication and helping her take it. According to her, this was one of the reasons why she had been able to cope with the disease. This reflects her statement:

*“Anytime I was visiting the hospital, my sister always accompanied me. She did all the queuing and rounds the nurses wanted me to do and helped me take my medication. I could not walk, so she really saved me.”* (Respondent 7).

Some respondents reported that their family and friends, as well as church members, prayed for them. According to them, they felt restored, revived and full of hope after the prayers. They believed that without the prayers, they could not have survived the treatment and the constant fear of death. The following were the responses of some respondents:

*“My friends and church members were also helping me with prayers”* (Respondent 4).

*“My husband was just praying. My children were also equally praying for me.”* (Respondent 12).

#### Financial support

Due to the cost of treatment and the inability of the women to work during treatment, some of the respondents needed financial support. According to them, they received financial support from their children, parents, other relatives, church members, spouses, relatives of their spouses, friends as well as neighbours. For some, their children paid their hospital bills, which were between GhC 300 ($72) and GhC 2000 ($477). Some respondents said:

*“My husband was helping me in financial aspect and the children and family also helped.”* (Respondent 8).

“*For the financial aspect, I was assisted by my sister and children. They paid my bills for me and even gave me some money to live on when I was in the hospital. They spent close to GhC 2000.00 ($477) on me”* (Respondent 4).

To them, they had financial support from their siblings whenever they had to go for treatment and any medical check-up. This has given them a boost during the treatment process of the disease.

#### Supporting services

Some respondents also recounted receiving some supporting services including washing and cooking by their children, relatives, relatives of their spouses, formal caregivers, friends, and self-help from other well-wishers. They explained that these relatives cooked for them, washed their clothes and cleaned their homes while they were at the hospital. For instance, one respondent in her narration noted:

*“When I came here, I was told there are people who cater for patients for a fee, so my child gave one of those people money. They charged GhC500 ($120) which she paid. Every now and then, they will come for money to buy me food and provisions. When my things get dirty, they will come and wash for me”* (Respondent 11).

Another respondent also had a similar support from her relatives, so she did not have to pay for it, and this reflected in her narration:

*“In terms of washing, cleaning, cooking, I was assisted by my relatives and my husband’s relatives”* (Respondent 13).

Respondents indicated that they received a number of supports from different sources and this had helped them to cope with their daily chores as well as the excruciating effects of the disease.

### Health seeking behaviour after diagnosis

Health seeking behaviours differed from one respondent to another. Respondents engaged in various health-seeking behaviours including orthodox medicine, herbal medicine, and faith healing.

#### Orthodox medicine

All the respondents reported ever using the orthodox medicine after diagnosis. They sought treatment from public hospitals such as the Battor Catholic Hospital, Korle-Bu Teaching Hospital, and the Ridge Hospital as well as some private hospitals. The Battor Catholic Hospital was the facility where most of the respondents were diagnosed, while a few were diagnosed at other private facilities. For those who required surgery, the staff at Battor Catholic Hospital provided assistance for them. Patients who required therapy were then referred to Korle-Bu Teaching hospital. During the treatment, respondents were admitted to the hospital for periods between one and 4 weeks. The respondents indicated that they were given drugs in the form of injections, intravenous fluids, and tablets. Some of them had undergone surgery while others had the cancerous sections removed.

*“The doctor told me that the womb will be removed through operation. He said they will fold my womb and cut it and then I will come and sleep in the hospital for one week or so. I went and they did it for me.”* (Respondent 6).

Some respondents also reported having gone through either chemotherapy, brachytherapy or radiotherapy in order to treat the condition. This reflects her statement:

*“I did radiotherapy and chemotherapy. Finally, we had to go for brachytherapy at Korle-Bu (a public hospital). The doctor said it will kill the cancer and I will be free.”* (Respondent 2).

Another respondent also made a similar statement concerning her treatment process. She explained:

*“When I came, I was asked to lie in a machine. I had to lie in this machine for three to six weeks. I cannot recall the exact number of weeks. I lay in the machine for some time and I was asked to take an injection.”* (Respondent 14).

When they were finally healed, the respondents had to go for regular reviews to monitor the progress of the treatment they had received. Some of them reported visiting the health facility every 2 weeks or every month. One respondent, therefore, expressed this in her narration:

*“After the operation, they had cleaned the place for me and after the sore is healed, I had to go for the review. I was going for review every month. I had to go for review so that the doctor will see that everything is in good condition and that I am actually doing well”.* (Respondent 12).

The respondents explained that they used orthodox medicine because they believed that that approach was essential for their survival. Specifically, they went to the hospital because the symptoms of the disease (such as pains, profuse bleeding, vaginal smell, and paleness), which they did not understand, were negatively affecting them and believed that they could not heal it by themselves.

#### Herbal medicine

Some respondents also reported using herbal medicine to manage the condition. They explained that they used herbal medicine because they felt that the orthodox medicine might fail or had not produced the expected results for them in the past. They applied herbal medicine to treat the disease, stop the blood flow and relieve the pain they experienced. These included local herbs, Chinese medicines or food supplements such as *Aloe vera*, ginger, moringa, dandelion, and garlic. Some even admitted combining local herbs, Chinese medicine, and food supplements. For instance, one respondent explained:

*“I went to the hospital, but unfortunately the bleeding became more, so I started looking for local medicines.”* (Respondent 2).

They, therefore, described the local medicines as home-made products from roots, barks, and leaves of trees. Some of these products can be rubbed on the stomach while others can be taken as syrups and tablets and are believed to be effective since they eventually stop the blood flow.

#### Faith healing

Some respondents also sought treatment from faith healers. According to them, it was God who heals. Some of them prayed for themselves and/or were prayed for by others. Those who went to church to seek divine intervention had hands laid on them or given anointing oils and holy water to drink. Following from this, one respondent had reported that she had to travel from Ghana to Nigeria to consult a renowned man of God (name withheld) for healing. She explained:

*“I decided to go to Nigeria and add prayers because God is the finisher of everything. I went and came back and the cancer was off. So this is how I went about the condition. I went the second time and it was all gone. So, really, with cancer, you cannot just treat it like that. You have to treat it with God because God is the ultimate healer. That man of God, I knew very well is a healer. It is the grace of God because at the hospital it was not easy for me at all.”* (Respondent 2).

A number of respondents also reported that in times of emotional or physical difficulties, they resorted to religion or divine intervention. They believed that if they turned to God, they would receive mercy and healing. Consequently, some decided to personally pray consistently for their healing. This reflects in the following report by a respondent:

*“I had to always pray to God. I just prayed to God so that he can do everything for me. Sometimes, I would be praying and ask God not to let me die. Sometimes too, I would be having some bad dreams and needed to pray. That is all I do to cope with the situation.”* (Respondent 12).

In short, the respondents believed that the various health-seeking behaviours they adopted helped them in a number of ways by suppressing the excruciating effects of the disease on their wellbeing. Examples are body pains, frequent blood flow, and inability to walk. All these helped them to easily cope with the disease. Some of them also believed that faith healing complemented the orthodox and herbal medical treatment in helping them to get cured totally. They, therefore, considered it quite effective for curing the disease and clearing it out of their system completely.

## Discussion

The study results revealed that cervical cancer patients employed numerous active and avoidant strategies to cope with their condition. For instance, some tried to forget about the condition while some have even denied the existence of the disease knowing that was a possible way of coping with the excruciating effects of the disease. Even though this may not be the best way to manage such a serious condition, the women believed that it was beneficial in seeing them through the treatment and recuperating stages. In a similar study among breast cancer patients, it was also observed that positive attitude or positive suggestion and re-affirmations were some of the major coping mechanisms adopted by the respondents [14]. In another study, breast cancer and cervical cancer patients resorted to positive thinking and purposeful lifestyle as a coping mechanism [15].

Furthermore, some respondents adopted abstinence from sexual intercourse throughout the treatment process to enable them to cope with their condition. Respondents indicated that to avoid worsening the disease condition and having a successful treatment, they had to give up sexual intercourse with their male partners throughout the treatment process until their condition had improved. A possible implication of this strategy is that it may encourage extramarital affairs on the part of the male partners. Also, cervical cancer patients used sanitary products including sanitary pads, cloths as well as disinfectants to manage the physical effects of the condition. Respondents used sanitary pads to quell continuous blood leakage. Some respondents further explained that using sanitary materials together with disinfectants made them feel hygienic and comfortable as well as helping to boost their self-image. As a result, they were more able to sit and take part in public activities than before. These aforementioned mechanisms may be considered as some of the several conscious and healthy living practices that people living with chronic diseases adopt in order to help them to alleviate the devastating effects of the disease. Büssing et al. [16], among cancer patients, also found that patients adopted other conscious and healthy living virtues in order to cope with their condition. It, therefore, becomes notable that conscious and healthy living practices have become indispensable for people living with chronic diseases including cancer patients in coping with their disease.

It has also been observed that various kinds of support were important sources of coping in times of chronic diseases [14, 15, 17]. Some cervical cancer patients were able to cope with the disease with the support of their partners in terms of social, emotional, financial, and physical support as well as supporting services. Other family members such as children, siblings, nieces, and nephews also provided some form of financial, social and emotional support. Ramanakumar et al. [15] have alluded to the importance of social support from other family members, friends as well as members of the community in helping patients to cope with unpleasant situations. The women, therefore, reported that the various supports provided by the different categories of people strengthened them in a number of ways and helped them to survive the disease. For instance, the family and community support helped patients by making them feel valued, and preventing social stigma, anxiety, fatigue and depression among the patients during the sick period. It also alleviated the stress and effects of overthinking and worrying that may be associated with the disease as well as saving patients from engaging in chores that may exacerbate their condition. The implication for health care is that social support is crucial in the treatment and survival of patients suffering from chronic diseases including cervical cancer. Also, this can be better promoted through consistent and effective health education.

The study also explored the health-seeking behaviour of the respondents as a means of managing the condition. In view of this, it came out that respondents used orthodox medicine, herbal medicine, and faith healing. However, these types of health-seeking behaviours have been sought in varied patterns. While some respondents sought faith healing first, some also initially went for orthodox medicine, and others went for herbal medicine and supplements to buttress orthodox medicine. The use of these modes of health care has been observed in a number of studies among Africans living with chronic diseases [18–20] and among Europeans [16].

From this study, it follows that the orthodox medicine largely had a positive effect on the coping abilities of the respondents. For instance, it was during the use of the orthodox medicine that the patients got knowledge about the stage of their disease and forms of treatment which set the pace for the entire coping process. However, the perceived effectiveness of herbal medicine by the women was mixed. While some reported improvement in their condition, some reported no change and others reported deterioration of their condition. Perhaps, three factors could explain the decision to seek herbal medicine. Firstly, the herbal medicine is without surgery and cheaper compared to orthodox medicine. Secondly, unavailability of drugs at the health facilities can also be a cause. There have been reports of lack of medication for cervical cancer treatment at health facilities in the country [21, 22]. Lastly, the lack of improvement that some respondents experienced after using orthodox medicine can also explain why they resorted to herbal medicine.

The health-seeking behaviour adopted by the patients had implications for treatment and adherence to the orthodox medicine. In particular, it has been found that the use of herbal medicines has no substantial improvement in destroying cancerous cells and relieving the pain associated with cervical cancer [23]. Besides, it has been reported that herbal medicine plays a major role in the complications and high mortality observed among patients [15]. Some respondents also used faith healing as a coping strategy [16]. For instance, most of them believed that if they honestly believed in God all their disease conditions would be solved. Consequently, some tried prayers and travelled to men of God to perform various religious activities for divine intervention. This is not unusual since research has established that people used religion to cope with their health problems [18, 24]. Mukwato et al. [14] also observed that reliance on God was one of the predominant mechanisms used by patients as well as their family members to manage difficult conditions such as breast cancer. Following from this, it becomes obvious that healthcare personnel working with faith leaders may have a positive implication for the health care of cervical cancer patients in this sub-region. One acknowledgeable limitation of the study lies in the fact that it was conducted in only one health facility. Nevertheless, the in-depth exploration of the subject provided an important insight into the real experiences of the cancer patients, which can inform interventions. Also, since the study setting is one of the few renowned cervical cancer screening and care facilities in the country, we believe that this study provided comprehensive information on the situation. However, future research could focus on extending the horizon of the study by including cervical cancer patients from other health facilities such as the Korle-Bu Teaching Hospital and the specialist private health facilities involved in cervical cancer care.

## Conclusions

Cervical cancer patients adopted various mechanisms to cope with the condition. They adopted personal and psychological coping strategies, social, financial and supporting services to manage the challenges associated with the disease. Health seeking behaviours in terms of seeking faith healing, herbal medicine and biomedicine also helped patients to manage the condition. It, therefore, became obvious that out of pains and frustration, some cervical cancer patients adopted inadequate and non-scientific approaches as a means of coping with the disease. Consequently, it is imperative for interventions targeted at cervical cancer patients to focus more on strengthening knowledge about the disease, financial, social and non-material support services as well as the use of screening and treatment health facilities.

## Abbreviations

GHS-ERC: Ghana Health Service Ethical Review Committee
HPV: Human Papilloma Virus
RQDA: R software package for Qualitative Data Analysis
